# *Achillea biebersteinni Afan* may inhibit scar formation: In vitro study

**DOI:** 10.1002/mgg3.640

**Published:** 2019-04-09

**Authors:** Maryam Hormozi, Parastoo Baharvand

**Affiliations:** ^1^ Razi Herbal Medicines Research Center Lorestan University of Medical Sciences Khorramabad Iran; ^2^ Department of Biochemistry Lorestan University of Medical Science Khorramabad Iran; ^3^ Department of Community Medicine Lorestan University of Medical Sciences Khorramabad Iran

**Keywords:** *Achillea biebersteinni Afan*, bFGF, fibroblast, scar formation, TGFβ1

## Abstract

**Background:**

One of the major problems in wound healing is scar formation; however, there are few ways to prevent or treat it. Different species of Achillea are used to treat wounds in folk medicine from the past but there are few studies on the effect of it on wound healing and inhibition of scar formation. The aim of this study was to investigate the effect of *Achillea biebersteinii Afan* hydroethanolic extract on the expression of TGFβ1 and bFGF as effective growth factors of wound healing in mouse embryonic fibroblast cells.

**Methods:**

Mouse embryonic fibroblast cells were exposed to different concentrations of Achillea extract at two different time (12 and 24 hr); the expression of TGFβ1 and bFGF was performed by real‐time‐PCR and ELISA at the level of gene and protein.

**Results:**

It was observed that the plant extract at 5 and 10 µg/ml downregulated the expression of TGFβ1 and upregulated the expression of bFGF at the level of gene and protein.

**Conclusion:**

The results showed that the pattern of changes in the expression of TGFβ1 and bFGF by *Achillea biebersteinni Afan* extract may inhibit scar formation.

## 1 INTRODUCTION

Scar formation is associated with permanent changes in normal tissue that is not desirable in the wound healing process. There are few strategies to prevent or to treat scarring that were not so successful (Lichtman, Otero‐Vinas, & Falanga, 2016). Growth factors have vital roles in this process, for example, it seems that overexpression of TGFβ1 may mediate fibrosis in wound while bFGF may promote scarless healing and reduce scarring in this process(Akita, Akino, & Hirano, 2013; Liu et al., 2016; Shah, Foreman, & Ferguson, 1995; Shi et al., 2013; Song, Bian, Lai, Chen, & Zhao, 2011; Wang et al., 2017). The usage of medicinal plants to treat wound has a long history; different effective components and biological balance of their compounds are sometimes more effective and have fewer side effects in treatment of wound, one of these plants is Achillea (Akkol, Koca, Pesin, & Yilmazer, 2011; Ghobadian et al., 2015; Khosra et al., 2017). Unfortunately, despite the historical records of the use of this plant in the treatment of wounds and injuries, there are few reports of its use for the treatment of wound.

*Achillea biebersteinni Afan* belongs to the Asterales family; different species of Achilea have different components such as tannins; volatile oils, mostly linalool, borneol, camphor, β‐caryophyllene, 1,8‐cineole, sesquiterpene lactones, flavonoids, amino acids, fatty acids, phenolic acids, vitamins, alkaloids, alkanes, polyacetylenes, saponins, sterols, sugars, and coumarins (Blumenthal, Goldberg, & Brinkmann, 2000).

Various studies have shown that this plant has antimicrobial, antioxidant, and anti‐inflammatory effects which are very important in the process of wound healing (Ali, Gopalakrishnan, & Venkatesalu, 2017; Stojanović, Radulović, Hashimoto, & Palić, 2005; Varasteh‐kojourian et al., 2017). The antimicrobial properties of this plant are due to compounds such as flavonoids, terpenes, hydroxycamptothecinand saponins (Mothana, Lindequist, Gruenert, & Bednarski, 2009; Stojanović et al., 2005; Yaghoubi, Gh, & Satari, 2007). Oxidant agents and free radicals are damaging agents to all stages of wound healing. They can damage to the fibroblasts and inhibit cellular messages by growth factors thus enhancing the antioxidant´s system playing an important role in accelerating wound healing. (Kurahashi & Fujii, 2015; Lisse, King, & Rieger, 2016; Loo, Ho, & Halliwell, 2011). It has been shown that flavonoids can inhibit the enzymes involved in the production of free oxygen radicals (cyclooxygenase, lipooxygenase, monooxygenase and glutathione S‐transferase) and chelate the metal ions resulting in antioxidant effects that have a crucial role in wound healing (Brown, Khodr, Hider, & Rice‐Evans, 1998; Ozcan, Korkmaz, Oter, & Coskun, 2005).The anti‐inflammatory effects of this plant are due to the effect of flavonoids on metabolism of arachidonic acid and the reduction of mitogenic cytokines such as gamma interferon, leukotrienes, prostaglandin E2, TNFα, and thromboxane B2 (Ozcan et al., 2005; Rotelli, Guardia, Juárez, De la Rocha, & Pelzer, 2003; Yauan, Wahlqvist, He, Yang, & Li, 2006).

Because there are no or a few studies reporting the effect of *A. biebersteinni Afan* on molecular levels so the aim of this study was to evaluate the effect *Achillea bibesteinii Afan* hydro‐acloholic extract on the expression of TGFβ1 and bFGF in the level of gene and protein in mouse embryonic fibroblasts.

## 2 MATERIAL AND METHODS

This research was an experimental study conducted at Razi Herbal Medicines Research Center with grant number: 2/91 in order to assess the potential effects of *A. biebersteinni Afan* extract on the induction of growth factors of wound healing in mouse embryonic fibroblasts.

### 2.1 Chemicals

SYBR‐Green quantitative polymerase chain reaction (q‐PCR) kit (Jena Bioscience, Germany), TGFβ1 (eBioscience, USA), and bFGF (RayBiotech, USA). Chemicals and biochemical reagents are of analytical grade and were freshly prepared and used.

### 2.2 Preparation of Achillea biebersteinni Afan flowers extract

*Achillea biebersteinni Afan* flowers were collected in the first half of July 2016 from Aligudarz area in Lorestan province, a mountainous place in west of Iran. The collected flower was authenticated at Razi Herbal Medicines Research Center, Lorestan University of Medical Sciences. For preparation of the extract, 20 g of dried flowers was soaked in 120 ml of 50% ethanol for 72 hr in the dark. It was centrifuged and passed through a filter, dried at 37°C. The yield of the extract was 8.8% w/w. Then the extract was stored at −20°C. Analysis of  the extract were performed by gas chromatography and mass spectrometry.

### 2.3 Isolation and culture of mouse embryonic fibroblasts

Isolation and culture of mouse embryonic fibrobalsts (MEFs) were performed according to the protocol of Jozefczuk et al. that previously was described (Hormozi, Assaei, & Boroujeni, 2017; Jozefczuk, Drews, & Adjaye, 2012). In brief, pregnant mouse was sacrificed 13 d.p.c (day postcoitum) by cervical dislocation. Uterine horns were dissected, rinsed with 70% (v/v) ethanol and soaked in PBS buffer w/o Ca/Mg ions. The experimental procedures were carried out under sterile conditions in a tissue culture hood. The uterine horns were placed in Petri dish and each embryo was separated from its embryonic sac and placenta. The tissue is chopped with carpels into smaller fragments, 1 ml of 0.05% trypsin/EDTA (Gibco, Invitrogen) containing 100 Kunits of DNase I was added to each embryo. The tissue was transferred into a 50 ml falcon tube and incubated for 15 min at the room temperature. Cells were dissociated every 5 min by pipetting thoroughly. Freshly prepared MEF medium was used for inactivating trypsin. The lysate was centrifuged at low speed (300× *g*) for 5 min, the cell pellet was discarded and supernatant was suspended in a fresh MEF medium. A number of cells equivalent to 3–4 embryos in each T150 (TPP) flask coated with 0.2% of bovine gelatin (Gelatin from bovine skin, type B, Sigma) were plated for 2 hr. At this time, the fibroblasts (passage 0) were the only cells capable of attachment to the gelatin‐coated flasks. The cells were allowed to reach 80%–90% confluency at this level; the P0 cells were trypsinized and stored at −70C for future usage. Other cells in the flask were allowed to reach P3 or P4 before use.

### 2.4 Scratch wound assays

A 24‐well tissue culture plate was coated with 0.2 mg/ml collagen type I through incubation (Sigma, Dorset UK) at 37°C for 2 hr, it was then washed with PBS (Invitrogen). Each of the well was seeded with cells (fibroblasts) to a final cell density of 100,000/well and these were maintained at 5% CO2, 37°C for 24 hr to allow for the attachment of cell and confluent monolayer formation. These were then scored using a sterile pipette tip to make a scratch of about 0.4–0.5 mm in width. Culture medium was then quickly removed (including any dislodged cells). The removed medium was then exchanged with a fresh serum‐free culture medium. All scratch assays were carried out in four replicates.

### 2.5 MTT assay for viability

MTT assay was performed to assess the cell viablity and proliferation of the extract. Briefly, cells were added at a density of 104 cells/well in DMEM with 10% FBS on micro culture plates. After 24 hr of incubation, cells were treated with different concentration of the extract (5, 10, 20, and 40 μg/ml) for 12, 24, 48, and 72 hr of treatment at 37°C and 5% CO_2_. Then 20 µl of MTT solution (5 mg/ml) was added to each well and after 4 hr of incubation at 37°C, the medium was removed and the purple colored formazan crystals were dissolved in 200 µl DMSO. The absorption of each well was measured using an ELISA reader at 570 nm. The reference wavelength was 690 nm. Percentage cell viability was obtained as follows:

$$\%\;\text{Cell}\;\text{viability}=\left(\text{OD}\;\text{treated}\;\text{cells}/\text{OD}\;\text{untreated}\;\text{cells}\right)\times 100.$$

### 2.6 Analysis of gene  and protein expression of TGFβ1 and bFGF

#### 2.6.1 TGFβ1 and bFGF gene expression analyses using real‐time PCR

RNA was extracted from untreated (control) and treated fibroblasts with different concentrations of *A. biebersteinni Afan* extract (5 and 10 μg/ml) for 12 and 24 hr using the total RNA purification kit (Jena Bioscience, Germany) according to the instructions in protocol kit and quantified using a BioPhotometer plus (Eppendorf, Hamburg, Germany). First‐strand cDNA was synthesized using the cDNA Synthesis Kit (Roche, Germany). Quantitative PCR (qPCR) was performed with Rotor‐Gene 6,000 and SYBR‐Green quantitative PCR (qPCR) kit (Jena Bioscience, Germany). The PCR conditions proceeded with an initial denaturation for 2 min at 95°C, 40 cycles with denaturation at 95°C for 15 s, primer annealing at 60°C for 40 s and primer extension at 72°C for 30 s and a final extension step at 72°C for 5 min. The sequences of the primers and their characteristics were previously presented^24^.

#### 2.6.2 TGFβ1 and bFGF protein extraction and expression analyses using ELISA

Cells were treated with different concentrations of extract of *A. biebersteinni Afan* (5 and 10 μg/ml) for 12 and 24 hr periods and the supernatants from treated and untreated (control) were collected. The cells were trypsinized, harvested in DPBS (pH 7.4), centrifuged, and then resuspended in RIPA buffer (Sigma Aldrich St. Louis, MO) with a freshly added protease inhibitor cocktail (1%) and phosphatase inhibitor cocktail (1%). Following the incubation at 40C for 45 min, the cell lysate was centrifuged at 14,000 rpm 40°C for 30 min for cell debris removal. TGFβ1 and bFGF protein concentrations were measured by ELISA kits (eBioscience and RayBiotech, USA) based on the kits protocols. In brief, samples were filled into 96‐well plate overnight with an appropriate dilution of capturing antibody in 0.2 M Na‐P buffer (pH6.5). The plate was rinsed three times using PBS plus 0.05% Tween‐20 (PBS‐T) and following this, it was blocked using Assay Diluent at 21°C for 60 min. For activating latent TGFβ1 and bFGF, samples were treated with 20 µL solution of 1N HCl/100 µl for 10 min and then it was neutralized with 1N NaOH. One hundred microliter of the sample, which was prepared in assay diluent, was added and incubated at 21°C for 2 hr. After washing five times with PBS‐T, 100 µl of assay diluent which contains Avidin‐horseradish peroxidase reagent and biotin‐conjugated detecting antibody at an appropriate dilution were added and incubated at 21°C for 60 min. After washing for seven times, 100 µl of the substrate solution was added to each well, incubated for 30 min in dark condition, and 50 µl of 2N H2SO4 was added to stop the reaction. The ELISA result was quickly read within 30 min by using an ELISA reader at 450 nm. The results were expressed as TGFβ1 and bFGF level in pg/ml of cell culture supernatant. The final calculation was carried out using a dilution factor of 1.4 to assess acid activation/neutralization.

### 2.7 Statistical analysis

In order to analyze the ratio of expression the genes of interest at two concentrations between treatment groups and untreated (control) group, a randomization test implemented in the relative expression software tool (REST) (Pfaffl, Horgan, & Dempfle, 2002) was used. Protein expression in the groups of treated and untreated was compared by Wilcoxon using SPSS software. Differences were considered significant at *p* < 0.05.

## 3 RESULTS

### 3.1 Phytochemicals in *A. biebersteinni Afan* extract

**Figure 1:**
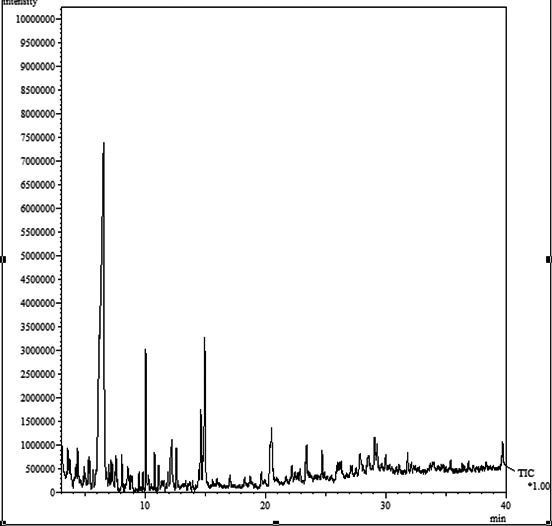
GC‐mass chromatogram for *Achillea biebersteinni Afan*

Following phytochemical screening *A. biebersteinni Afan* extract is said to be rich in hydroxycamptothecin (HCPT). HCPT is noted as one the most potent agents against scars, it inhibits the proliferation of fibroblast and decreases the adhesion of epidural (Figure  1).

### 3.2 Scratch assay

**Figure 2:**
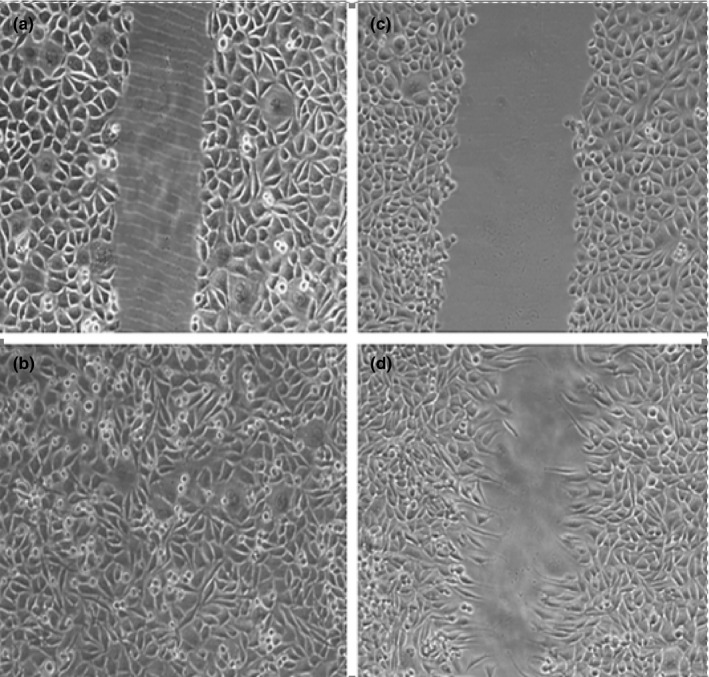
Image of *Achillea biebersteinni Afan* enhanced rate of wound closure in fibroblasts. (a) scratching effect on fibroblast cells at 0hrs (b) The healing effect of the extract on fibroblast cell (c) control group at 0 hr (d) control group at 9 hr. Data are represented as means ± *SEM*, *p < 0.05

The results of scratch assay showed that A.biebersteinni Afan can decrease cell migration (Figure 2).

### 3.3 Effect of *A. biebersteinni Afan* extract on cell viability

**Figure 3:**
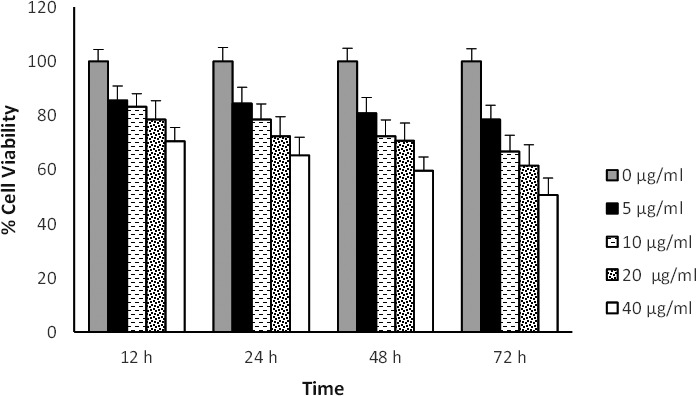
Effect of various concentrations of Achillea on mouse embryonic fibroblast cell viability. Cells were treated with different concentration of Achillea for 12, 24, 48 and 72 hr and cell viability was measured by MTT assay

The results of MTT assay showed that extract of *A. biebersteinni Afan* can reduce the cell viability of fibroblasts (Figure 3). Based on MTT results, concentration of 5, 10 μg/ml of A.biebersteinni Afan and times of 12, 24 hr were selected for treatment. So the expression of growth factors were evaluated in these concentrations and times (Figure 3).

### 3.4 TGFβ1 and bFGF gene expression

**Figure 4:**
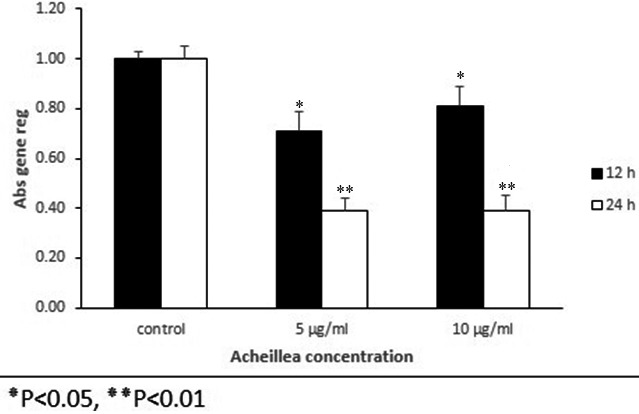
Relative expression of TGFβ1 gene in mouse embryonic fibroblast cell that were treated with various concentration of Achillea compared to control group at different time intervals treatment (12 and 24 hr). Mouse embryonic fibroblast cells were treated with Achillea (5 and 10 µ) and expression of TGFβ1 gene was assessed by quantitative real time PCR. All comparisons were compared to the control group (*p* < 0.05)

**Figure 5:**
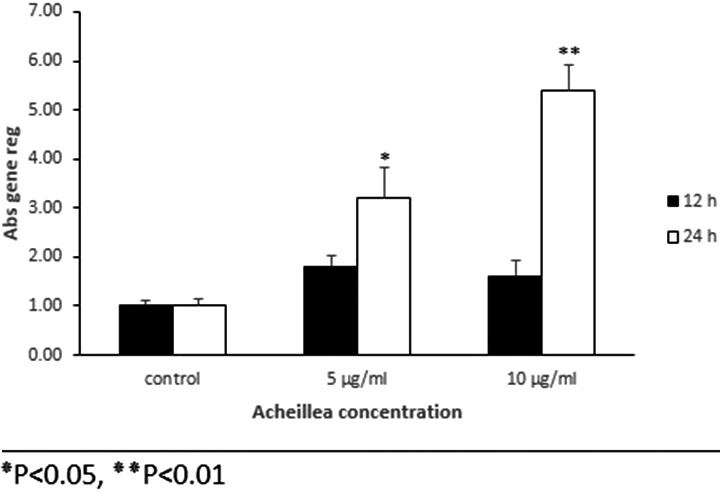
Relative expression of bFGF gene in mouse embryonic fibroblast cell that were treated with various concentration of Achillea compared to control group at different time intervals treatment (12 and 24 hr). Mouse embryonic fibroblast cells were treated with Achillea (5 and 10 µ) and the expression of bFGF gene was assessed by quantitative real time PCR. All comparisons were compared to the control group (*p* < 0.05)

The results of gene expression for *TGFβ1* in treated groups compared to the control group indicated a decrease in gene expression after 12 hr but this reduction was significant after 24 hr for both concentrations (Figure 4). The gene expression of *bFGF* in treated cells compared to control showed opposite patterns with *TGFβ1* expression (Figure 5).

### 3.5 TGFβ1 and bFGF protein expression

**Figure 6:**
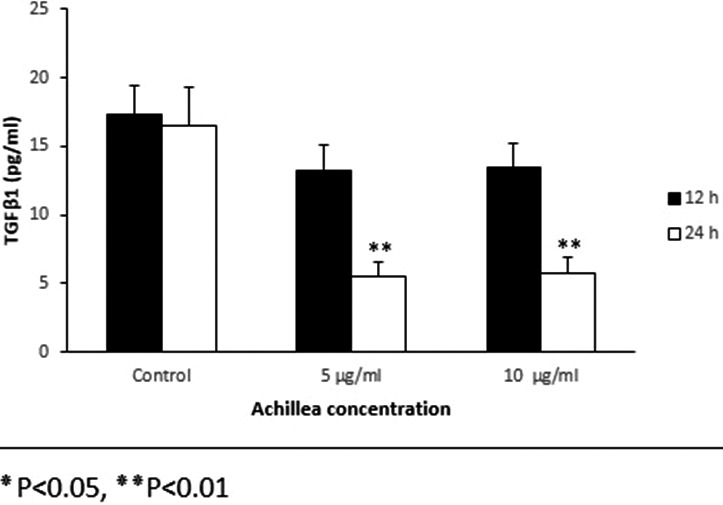
Effect of various concentrations of Achillea on the expression of TGFβ1 protein in mouse fibroblast cell culture supernatants. Cells were treated with different concentration of Achillea (5 and 10 µg/ml) at different time intervals treatment (12 and hours) and the expression of TGFβ1 protein was assessed by ELISA (*p < 0.05, **p < 0.01)

**Figure 7:**
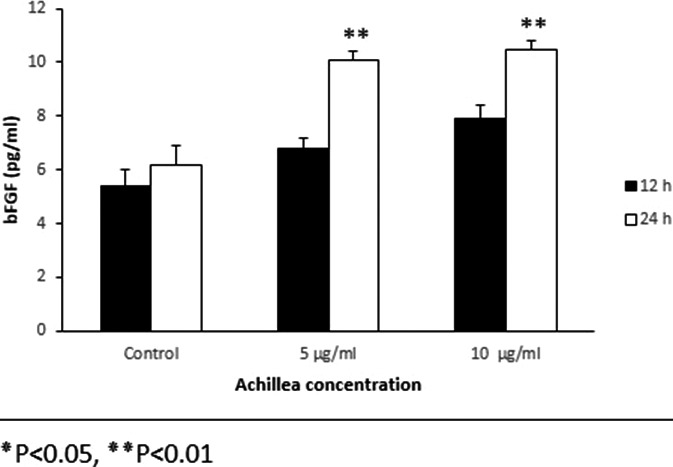
Effect of various concentrations of Achillea on the expression of bFGF protein in mouse fibroblast cell culture supernatants. Cells were treated with different concentration of Achillea (5 and 10 µg/ml) at different time intervals treatment (12 hr) and the expression of bFGF protein was assessed by ELISA (*p < 0.05, **p < 0.01)

The analysis of proteins expression showed that the expression of protein of TGFβ1 was decreased in the fibroblasts at different concentrations, compared to the control this reduction was significant after 24 hr (Figure 5). The protein expression of bFGF in treated cells compared to control showed an increase that was significant after 24 hr of treatment (Figure 6). In general, the pattern of expression of the protein was similar to the gene expression pattern (Figure 7).

## 4 DISCUSSION

Scar formation is one of the major issues in wound healing and fibrosis, therefore, to prevent or to treat scarring is an important aim in the medicine field. Fibroblasts play an important role in the process of wound healing by producing an extracellular matrix such as collagen, fibronectin, elastin acids, and differentiating into myofibroblasts but their hyperactivity destroys the balance between different processes in wound healing (Kendall & Feghali‐Bostwick, 2014; Song et al., 2011).

To address whether the extract can induce stimulated skin cell proliferation or survival in addition to cell migration during the closure of wound, fibroblast cell culture was established in a 24‐well plate in fibroblast versus serum‐free control medium. The MTS assay was then carried out to confirm the viable cell number. There was a nonsignificant, but marked increase in the number of cells at 9 hr in fibroblast when compared to the control (Figure 1).

Different studies have shown the role of cytokines and growth factors in this process. It seems that TGFβ1 and bFGF are more important than others. Although TGFβ1 has an important role in wound healing by cell proliferation, stimulating angiogenesis, granulation tissue formation, re‐epithelization, extra‐cellular matrix deposition, and remodeling but its excessive production in the wound site can cause scarring. Studies have shown that this factor increases proliferation of fibroblastic transformation into myofibroblasts and thus increases the formation of extracellular matrix. It seems that the mechanism of the effect of this factor is through the increase of the expression of micro R 21 and through PTEN/Akt pathway. It has also been shown that this factor inhibits the pathway of NOTCH/Jagged1 and so causes scar inhibition (Jagadeesan & Bayat, 2007; Lichtman et al., 2016; Liu et al., 2016; Penn, Grobbelaar, & Rolfe, 2012). Therefore, reducing TGFβ1 can be a strategy to prevent scarring and the same has been suggested by various studies.

Unlike TGFβ1 several studies have shown antiscar effects of bFGF on wound healing. It seems that the antiscarring effects of bFGF are due to reducing collagen and fibronectin and induction of the expression of the matrix metalloproteinases which leads to the reduction of the extracellular matrix, inhibition of the differentiation of fibroblasts into myofibroblasts, reduction of the level of ATP in the mitochondria and inducing apoptosis of the hypertrophic fibroblasts by activating Notch1/Jagged1 pathway (Akita, 2010; Grazul‐Bilska et al., 2003; Ono et al., 2007; Shi et al., 2013; Tiede et al., 2009; Wang et al., 2017; Xie et al., 2008; Zhu, Ding, & Tredget, 2016).

Although several studies have shown the effect of different species of Achillea on wound healing because of its antimicrobial and antioxidant properties, based on our knowledge there is no report on the antiscarring effect of this plant(Akkol et al., 2011; Dorjsembe et al., 2017; Khosra et al., 2017; Stojanović et al., 2005; Varasteh‐kojourian et al., 2017).

The proliferation and transdifferentiation of fibroblasts into myofibroblasts is one of the important mechanisms of scar formation, since the results showed that the extract of *A. biebersteinni Afan* can reduce the viability fibroblasts, it seems this plant can inhibit scar formation. The results also showed that the Achillea extract can reduce and increase the expression of TGFβ1 and bFGF, respectively. It seems that this pattern changes of growth factors may be intended to inhibit scar formation. Although more studies are needed on other growth factors that are effective in wound healing, especially in vivo models, to confirm the antiscarring effect of this plant.

In conclusion, pattern changes of growth factors of TGFβ1 and bFGF by *A. biebersteinni Afan* extract in fibroblasts may induce wound healing and reduce scar formation.

## CONFLICT OF INTEREST

There is no conflict of interest in this paper.
